# Radiomics Analysis of MR Imaging with Gd-EOB-DTPA for Preoperative Prediction of Microvascular Invasion in Hepatocellular Carcinoma: Investigation and Comparison of Different Hepatobiliary Phase Delay Times

**DOI:** 10.1155/2021/6685723

**Published:** 2021-01-07

**Authors:** Shuai Zhang, Guizhi Xu, Chongfeng Duan, Xiaoming Zhou, Xin Wang, Haiyang Yu, Lan Yu, Zhiming Li, Yuanxiang Gao, Ruirui Zhao, Linlin Jiao, Gang Wang

**Affiliations:** ^1^Department of Radiology, The Affiliated Hospital of Qingdao University, Qingdao, China; ^2^Department of Radiology, Zhucheng People Hospital, Zhucheng Shandong, China; ^3^Operating Room, The Affiliated Hospital of Qingdao University, Qingdao, China; ^4^Interventional department, The Affiliated Hospital of Qingdao University, Qingdao, China

## Abstract

**Purpose:**

To investigate whether the radiomics analysis of MR imaging in the hepatobiliary phase (HBP) can be used to predict microvascular invasion (MVI) in patients with hepatocellular carcinoma (HCC).

**Method:**

A total of 130 patients with HCC, including 80 MVI-positive patients and 50 MVI-negative patients, who underwent MR imaging with Gd-EOB-DTPA were enrolled. Least absolute shrinkage and selection operator (LASSO) regression was applied to select radiomics parameters derived from MR images obtained in the HBP 5 min, 10 min, and 15 min images. The selected features at each phase were adopted into support vector machine (SVM) classifiers to establish models. Multiple comparisons of the AUCs at each phase were performed by the Delong test. The decision curve analysis (DCA) was used to analyze the classification of MVI-positive and MVI-negative patients.

**Results:**

The most predictive features between MVI-positive and MVI-negative patients included 9, 8, and 14 radiomics parameters on HBP 5 min, 10 min, and 15 min images, respectively. A model incorporating the selected features produced an AUC of 0.685, 0.718, and 0.795 on HBP 5 min, 10 min, and 15 min images, respectively. The predictive model for HBP 5 min, 10 min and 15 min showed no significant difference by the Delong test. DCA indicated that the predictive model for HBP 15 min outperformed the models for HBP 5 min and 10 min.

**Conclusions:**

Radiomics parameters in the HBP can be used to predict MVI, with the HBP 15 min model having the best differential diagnosis ability.

## 1. Introduction

Hepatocellular carcinoma (HCC) is one of the most common malignant tumors in the liver [1]. Surgery is regarded as the first choice for eligible patients [2]. Microvascular invasion (MVI) is a vital predictor of HCC recurrence, especially in the early stage after surgical resection [3, 4]. Previous studies have identified MVI as a major risk factor for early recurrence within two years after hepatectomy and transplantation [5]. The application of preoperative imaging methods to predict MVI has important clinical significance. Therefore, it is necessary to predict MVI to identify tumor invasion and predict tumor recurrence after hepatectomy and transplantation.

Previous studies found that some imaging features, such as the tumor size, shape, capsule, margin, apparent diffusion coefficient (ADC) values, and enhancement pattern, may contribute to the diagnosis of MVI before surgery [6–8]. However, these qualitative findings can be affected by many factors, including the variability between observers and the lack of external validation, and there is still debate about the predictive value of MVI in HCC. Recently, radiomics analysis has become an emerging quantitative image processing method. It can quantify tissue heterogeneity by evaluating the distribution of radiomics roughness and irregularity within lesions. Different from tissue biomarkers, which can assess the microheterogeneity of regional tumors, radioactive biomarkers can noninvasively examine the whole tumor at the millimeter level [9]. Therefore, this method is expected to quantitatively evaluate lesion characteristics in more detail and with better repeatability than visual analysis by human observers. Some published studies have evaluated the potential of radiomics in predicting MVI in hepatocellular carcinoma [4, 10–12]. To the best of our knowledge, no research on predicting MVI or comparing imaging at different hepatobiliary phase (HBP) times using radiomics analysis of gadolinium-ethoxybenzyl-diethylenetriaminepentaacetic acid- (Gd-EOB-DTPA-) enhanced MR has been reported.

Thus, the aim of this study was to investigate whether radiomics analysis of MR imaging with Gd-EOB-DTPA in HBP can be used to predict MVI in patients with HCC and compare the prediction of MVI on different HBP delay times.

## 2. Materials and Methods

### 2.1. Patients

This retrospective study was approved by our institutional review committee, and patient informed consent was waived. By searching our institution's database, 294 consecutive liver cancer patients were selected between January 2015 and May 2020. The inclusion criteria were as follows: (1) MR images showing liver tumors larger than 1 cm in diameter; (2) Gd-EOB-DTPA-enhanced MRI scan including complete examination recordings at HBP 5 min, 10 min, and 15 min; and (3) HCC diagnosed by postoperative pathology. The exclusion criteria were as follows: (1) patients who underwent MRI examination more than one month before surgery; (2) patients who had received liver cancer treatment before surgery; and (3) insufficient image quality for radiomics analysis. Finally, 130 HCC patients, including 80 MVI-positive patients and 50 MVI-negative patients, were included in this study. The MVI information was obtained from the HIS system at our hospital and was diagnosed by the same pathologist. According to the date of MRI, the cohort was divided into a training set (*n* = 91; 60 men and 31 women; mean age 57.8 ± 12.6 years) and a time-independent validation set (*n* = 39; 29 men and 10 women; average age 58.6 ± 11.6 years).

### 2.2. MR Techniques

All study patients underwent MR imaging using a 3.0T scanner (GEHCGEHC, GE medical systems, Waukesha, WI). A dose of 0.1 mL/kg (0.025 mmol/kg) Gd-EOB-DTPA (Primovist, Bayer HealthCare, Berlin, Germany)) was administered at a flow rate of 1.0 mL/s followed by 25 mL of saline. A 3D fat-suppressed Liver Acquisition with Volumetric Acceleration (LAVA, GE Healthcare) sequence was performed in the axial plane at 5, 10, and 15 min after contrast agent injection (HBP 5 min, 10 min, and 15 min, respectively). The imaging parameters of the LAVA sequence were as follows: TR/TE, 2.5/1.1; inversion time, 5.0 milliseconds; flip angle, 9°; thickness, 5 mm; slice spacing, 2.5 mm; FOV, 380–450 mm; 256 × 256 matrix; number of signals acquired, 0.70; and bandwidth, 976.6 kHz. The comparison of dynamic T1-weighted and T2-weighted imaging was not the focus of this study and was not conducted.

### 2.3. MR Radiomics Analysis

**Figure 1:**
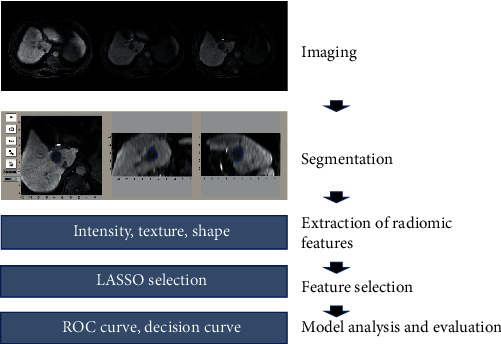
Workflow of radiomics analysis.

The workflow of the radiomics analysis included tumor segmentation, feature extraction, feature selection, and model construction and evaluation (Figure 1).

Three-dimensional segmentation of HCC using the IBEX software (http://bit.ly/IBEX) was performed by two radiologists in abdominal diagnostics with 8-year and 10-year MR experience who were blinded to the MVI information. When patients had multiple tumors, the largest tumor was analyzed. The regions of interest were drawn manually on HBP 5 min, 10 min, and 15 min images, covering the whole tumor. Radiomics parameters were selected using the IBEX software and included eight categories: Gradient Orient Histogram, Gray Level Cooccurrence Matrix 25, Gray Level Run Length Matrix 25, Intensity Direct, Intensity Histogram, Intensity Histogram Gauss Fit, Neighbor Intensity Difference 25, and Shape. Each category included different radiomics parameters. The intraclass correlation coefficient (ICC) of 30 randomly selected tumors was calculated to test the repeatability of features extracted by repeated segmentation, and features with an ICC less than 0.80 were excluded.

### 2.4. Statistical Analysis

Two independent sample *t* tests were used to compare the mean age between the MVI-positive and MVI-negative patients. The chi-square test was used to compare the sex distribution between the MVI-positive and MVI-negative patients. The least absolute shrinkage and selection operator (LASSO) regression method was used to select the most valuable parameter from all parameters obtained at HBP 5 min, 10 min, and 15 min. Receiver operating characteristic (ROC) curves and areas under the ROC curve (AUCs) of the radiomics parameters selected at each phase were calculated. The selected features at each phase were adopted into support vector machine (SVM) classifiers to establish models. SVM models were evaluated by 10-fold cross-validation to reduce overfitting. Multiple comparisons of the AUCs at each phase were performed by the Delong test with Bonferroni-adjusted *p* values. To present the distribution of the radiomics parameters in which HBP imaging can best differentiate MVI-positive and MVI-negative patients, a heat map was created. Decision curve analysis (DCA) was used to analyze the classification. The interobserver reproducibility of the selected valuable radiomics parameter was evaluated by ICC. SPSS 22.0 (Chicago, Illinois, USA) was used for statistical analysis. LASSO regression, ROC curves, the Delong test, and DCA were performed by using R (https://www.r-project.org/). *p* < 0.05 was considered statistically significant.

## 3. Results

### 3.1. Demographics

**Figure 2:**
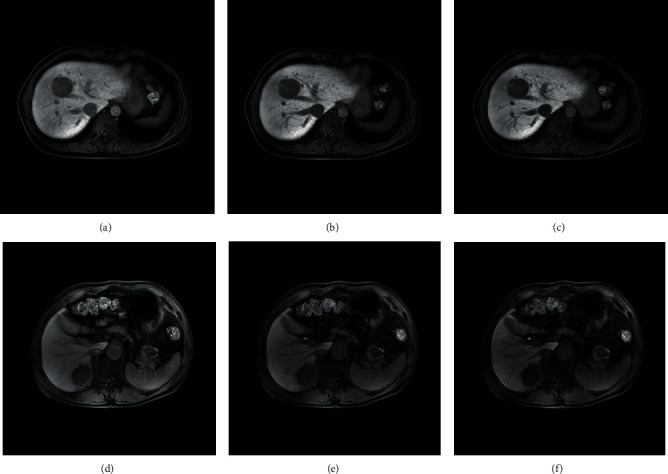
Axial MR imaging with Gd-EOB-DTPA on HBP in a HCC MVI-negative patient ((a) HBP 5 min, (b) HBP 10 min, and (c) HBP 15 min), and a MVI-positive patient ((d) HBP 5 min, (e) HBP 10 min, and (f) HBP 15 min). The imaging of MVI negative shows a smooth tumor margin, while MVI-positive shows a nonsmooth tumor margin. However, other tumor features between MVI positive and negative are difficult to identify by visual inspection.

Eighty MVI-positive HCC patients and 50 MVI-negative patients were included. There was no significant difference in age or sex between MVI-positive and MVI-negative patients. Examples of HCCs in MVI-positive and MVI-negative patients are shown in Figure 2.

### 3.2. Comparison of MR Radiomics Analyses with LASSO Regression

**Figure 3:**
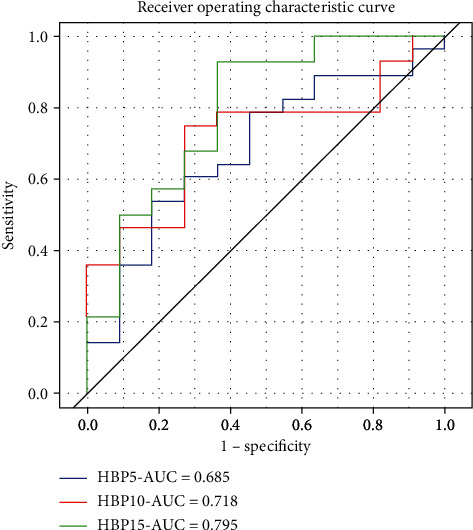
The ROC curves and AUC values of HBP 5 min, 10 min, and 15 min to differentiate MVI-positive and MVI-negative patients. The HBP 15 min produced the highest AUC of 0.795.

Each ROI has 8 categories and 1768 radiomics parameters. The most predictive features between MVI-positive and MVI-negative patients included 9 radiomics parameters at HBP 5 min, 8 radiomics parameters at HBP 10 min, and 14 radiomics parameters at HBP 15 min (Table 1). The two radiomics parameters with the top two AUC values were X0.7 Homogeneity (AUC = 0.641) and Compactness2 (AUC = 0.615) in the hepatobiliary phase (HBP) at 5 min, X1.7 Contrast (AUC = 0.625) and X4.7 Auto Correlation (AUC = 0.605) in the hepatobiliary phase (HBP) at 10 min, and X6.1 Difference Entropy (AUC = 0.645) and X4.7 Dissimilarity (AUC = 0.638) in the hepatobiliary phase (HBP) at 15 min. A model incorporating all radiomics parameters selected by LASSO in each phase produced AUCs of 0.685, 0.718, and 0.795 at HBP 5 min, 10 min, and 15 min, respectively (Figure 3).

### 3.3. Comparison of the 3 HBP Delays in Differentiating MVI

**Figure 4:**
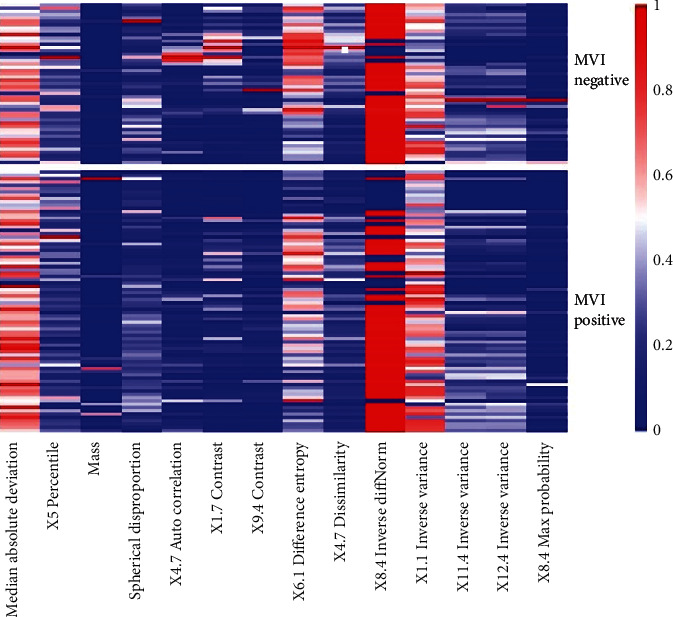
The heat map of HBP 15 min shows the distribution of the most predictive texture parameters between MVI-positive and MVI-negative patients. Difference in colors means different values of radiomics parameter.

**Figure 5:**
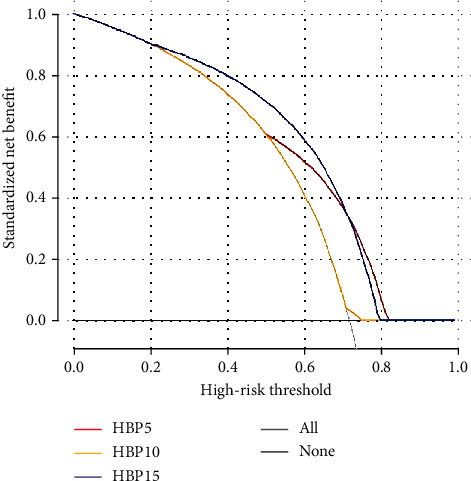
Decision curve analysis of HBP 5 min, 10 min, and 15 min. The *X* axis represents the threshold probability, and the *Y* axis represents the net benefit. HBP 5 min had no net benefit when the threshold probability is less than about 0.5. HBP 10 min had no net benefit almost across the range of the threshold probability. HBP 15 min had a larger net benefit than HBP 5 min when the threshold probability is less than about 0.7, and a little lesser net benefit when the threshold probability is about between 0.7 and 0.8.

The results of the Delong test used to differentiate MVI-positive and MVI-negative patients for the 3 HBP delays are shown in Table 2. The predictive model for HBP 5 min, 10 min, and 15 min showed no significant difference (HBP 5 min vs. HBP 10 min, *p* = 0.751; HBP 5 min vs. HBP 15 min, *p* = 0.362; HBP 10 min vs. HBP 15 min, *p* = 0.440). The radiomics parameter distribution at HBP 15 min is demonstrated with a heat map in Figure 4. The results of DCA at HBP 5 min, 10 min, and 15 min are shown in Figure 5. There was no net benefit of HBP 5 min when the threshold probability was less than approximately 0.5 and no net benefit of HBP 10 min within almost the same threshold probability range. HBP 15 min had a larger net benefit than HBP 5 min when the threshold probability was less than approximately 0.7, and there was a slightly lesser net benefit when the threshold probability was between approximately 0.7 and 0.8.

### 3.4. Interobserver Agreement for the Selected Valuable Radiomics Parameter at HBP 15 Min

The interobserver agreement between the 2 radiologists was good for the selected valuable radiomics parameter at HBP 15 min (ICC range: 0801–0.997) (Table 3).

## 4. Discussion

MVI is a vital independent predictor of early recurrence in HCC patients [13, 14]. Gd-EOB-DTPA is a biphasic T1-weighted MRI contrast agent which enters hepatocytes in an ATP-dependent manner through the organic anion transport polypeptide (OATP1B1/B3) and is finally excreted through the biliary tract. It is used for dynamically contrast-enhanced MRI of the liver, as well as the specific imaging process during the HBP after injection. A previous study indicated that radiomics signatures on HBP 20 min images could assess MVI in patients with HCC [15]. However, few studies have been conducted to assess MVI and compare HBP 5 min, 10 min, and 15 min images using radiomics from Gd-EOB-DTPA-enhanced MR. In the present study, after recruiting patients with HCC, we employed radiomics to assess MVI in HCC with Gd-EOB-DTPA on HBP 5 min, 10 min, and 15 min images. We verified the capability of the radiomics model for preoperative prediction of MVI status in a verification cohort.

Manifestations on the HBP images of Gd-EOB-DTPA-enhanced MRI indicate the functions of hepatocytes. HCC cells, relative to hepatocytes, fail to carry out the absorption of Gd-EOB-DTPA in the HBP. This can lead to low intensity within the tumor at this stage. However, previous studies [7, 16] reported that the occurrence of MVI cannot be predicted by assessing the difference in the occurrence of intratumoral hypointensity on HBP. In the present study, a model incorporating the radiomics parameters on HBP 5 min, 10 min, and 15 min images produced AUCs of 0.685, 0.718, and 0.795, indicating that the HBP model can assess MVI in HCC. This is because radiomics has the advantages of stable calculation, high repeatability, indefatigability, and being free from human subjective initiative interference [17, 18]. Tumor heterogeneity is likely to be difficult to identify and quantify by conventional imaging tools, the subjective assessment of images, or random sampling biopsy [19], whereas the mentioned techniques have been shown to be tightly associated with the pathophysiology of cancer. Existing studies have reported that the characteristics of radiomics show tight associations with the microstructure and biological behavior of tumors [20, 21]. In the present study, 14 quantitative characteristics on HBP 15 min images were found, which were not presented previously. Radiomics characteristics are important markers of intratumoral homogeneity. Of the 14 radiomics characteristics related to MVI in the present study, 2 were histogram-related characteristics (Median Absolute Deviation, 5th Percentile), 2 were shape-related characteristics (Mass, Spherical Disproportion), and others were matrix-related characteristics (4.7 Auto Correlation, 1.7 Contrast, 9.4 Contrast, 6.1 Difference Entropy, 4.7 Dissimilarity, 8.4 Inverse Diff Norm, 1.1 Inverse Variance, 11.4 Inverse Variance, 12.4 Inverse Variance, and 8.4 Max Probability). The features based on the histogram are first-order statistics, primarily determined by the statistics of intensity information (or brightness information) in and around the tumor. Subsequently, the overall distribution of intensity information in and around the tumor was explored. The signal intensity of MVI-positive HCC was lower than that of MVI-negative HCC, and differences in histogram characteristics were more frequent [22]. Shape-related characteristics were adopted to express the complexity of the lesion shape. Given histological studies, MVI-positive HCC exhibited an aggressive tendency, invading the tumor envelope and extending into the noncancerous substance, thereby causing a higher incidence of irregular tumor margins [23]. Matrix-based characteristics are second-order statistics applied to express lesions complex characteristics, the variation of hierarchical structure, and the thickness of texture. The difference in the mentioned parameters may indicate the heterogeneity of the tumor that is difficult to identify by the subjective assessment of images. Although radiomics has already been applied, it can effectively mark images, which can facilitate the assessment and quantification of processes of tumor space-related heterogeneity [24]. Nevertheless, the radiomics characteristics are acquired and determined with a PC. It is very challenging to explain the relationships between the radiomics characteristics, and pathology-related manifesting data are a challenge to develop [25]. First, the pathophysiological process involves several interacting parts; second, the maximum data acquired by the PC image study are significantly greater that acquired by visual examination.

The predictive models for HBP 5 min, 10 min, and 15 min had no significant differences according to the Delong test (HBP 5 min vs. HBP 10 min, *p* = 0.751; HBP 5 min vs. HBP 15 min, *p* = 0.362; and HBP 10 min vs. HBP 15 min, *p* = 0.440). To further compare the models for HBP 5 min, 10 min, and 15 min, this study applied DCA, i.e., a method to assess the models in terms of the clinical consequences and calculate the benefit and the loss of the assessed models for respective individuals [26]. This method attempts to overcome the limitations of traditional statistical indicators and complete decision analysis methods, which cannot directly provide clinical value information, nor can they be used in routine biostatistics practice [27]. The present study revealed that HBP 15 min images achieved the largest net benefit under the threshold probability, only with a slightly lesser net benefit when the threshold probability was between approximately 0.7 and 0.8. Gd-EOB-DTPA enters hepatocytes through organic anion transport polypeptides and is finally excreted through the biliary tract; this process takes some time to complete. Therefore, we predicted that this, in theory, is why the HBP 15 min model outperformed the HBP 5 min and 10 min model. Wu et al. [28] found that the severity of liver cirrhosis had a significant negative effect on the detection of HCC by HBP. For patients with severe cirrhosis, HBP 15 min or longer seems to be more suitable for HCC than BHP 5 min and 10 min. Nakamura [29] reported that more focal liver lesions could be assessed on HBP 15 min images compared with HBP 5 and 10 min images. HCC patients often have a background of cirrhosis, leading to varying degrees of damage to liver function. The present study showed that the predictive model for HBP 15 min outperformed the HBP 5 min and 10 min models, which is in line with the proposed theory and previous research. Feng et al. [4] reported that the AUC of the HBP 20 min model for predicting MVI in the training and validation cohorts was 0.85 and 0.83, respectively; the diagnostic efficiency of this model was slightly higher than that of our study. However, their model combined intratumoral and peritumoral radiomics information. Liang et al. [30] reported that HBP 15 min was sufficient for lesion characterization in cirrhosis patients with mild liver dysfunction when compared with HBP 20 min. Though the present study did not include data for HBP 20 min, we predicted that the model for HBP 15 min was sufficient for MVI prediction in HCC. Additionally, other features and biomarkers could be incorporated in HBP 15 min to improve diagnostic efficiency.

Several limitations are revealed in this study. First, this study was a retrospective study, which may have caused inevitable selection bias, and lacks external validation. Second, compared with the relatively large number of variables, the sample size remained limited. Third, our verification cohort and training cohort were from the same center, and the radiology analysis conducted for the stability assessment will be further optimized in future multicenter studies. Fourth, in the present study, only MR images of HBP at 5 min, 10 min, and 15 min were explored. There are no data for HBP at 20 min on account of daily busy clinical work pressure. A multicenter and prospective study with a longer delay time and a larger population is needed to validate these results in the future. Ideally, the characterization of MVI should involve both intratumoral and peritumoral areas; therefore, it was another limitation for only analyzing intratumoral area in this study.

## 5. Conclusion

In conclusion, radiomics parameters on the HBP 5 min, 10 min, and 15 min images after Gd-EOB-DTPA injection can be used to predict MVI for HCC, with the HBP 15 min model having the best differential diagnosis ability; this model has potential clinical value for preoperative noninvasive prediction of MVI in HCC patients.

## Figures and Tables

**Table 1:** The most predictive features between MVI-positive and MVI-negative selected by LASSO regression.

Phase	Radiomics parameter	Which category belongs to	Regression coefficient
HBP 5 min	Compactness2	Shape	0.30551482
	Mass	Shape	0.03586056
	VoxelSize	Shape	0.08370105
	TextureStrength	NeighborIntensityDifference25	-0.4756972
	7.7Energy	GrayLevelCooccurenceMatrix3	-0.22185
	0.7Homogeneity	GrayLevelCooccurenceMatrix3	0.17812152
	4.1InformationMeasureCorr1	GrayLevelCooccurenceMatrix3	0.10846544
	1.4InverseDiffMomentNorm	GrayLevelCooccurenceMatrix3	0.0502487
	X10.4InverseDiffNorm	GrayLevelCooccurenceMatrix3	-0.10842471

HBP 10 min	.333ShortRunHighGrayLevelEmpha	GrayLevelRunLengthMatrix25	-0.025523039
	0ShortRunHighGrayLevelEmpha -	GrayLevelRunLengthMatrix25	-0.037606409
	NumberOfObjects	Shape	-0.150641355
	SurfaceArea	SurfaceArea	0.04154656
	1.7Contrast	GrayLevelCooccurenceMatrix3	-0.327361605
	.333.7Dissimilarity	7Dissimilarity	-0.159344876
	7.7Energy	GrayLevelCooccurenceMatrix3	-0.247759079
	6.7MaxProbability	GrayLevelCooccurenceMatrix3	-0.301320203

HBP 15 min	MedianAbsoluteDeviation	GradientOrientHistogram	0.012259179
	5Percentile	GradientOrientHistogram	-0.064760131
	Mass	Shape	0.052851135
	SphericalDisproportion	Shape	-0.067929609
	4.7AutoCorrelation	GrayLevelCooccurenceMatrix3	-0.182481531
	1.7Contrast	GrayLevelCooccurenceMatrix3	-0.049683698
	9.4Contrast	GrayLevelCooccurenceMatrix3	-0.300911891
	6.1DifferenceEntropy	GrayLevelCooccurenceMatrix3	-0.105303216
	4.7Dissimilarity	GrayLevelCooccurenceMatrix3	-0.205762541
	8.4InverseDiffNorm	GrayLevelCooccurenceMatrix3	-0.002903426
	1.1InverseVariance	GrayLevelCooccurenceMatrix3	0.046277632
	11.4InverseVariance	GrayLevelCooccurenceMatrix3	-0.001397914
	12.4InverseVariance	GrayLevelCooccurenceMatrix3	-0.188965726
	8.4MaxProbability	GrayLevelCooccurenceMatrix3	-0.251615445

HBP, hepatobiliary phases.

**Table 2:** The results of multiple comparisons of the AUCs by the Delong test.

	*Z* statistic	*p*
HBP 5 min-HBP 10 min	-0.3173	0.751
HBP 5 min-HBP 15 min	-0.9121	0.362
HBP10min-HBP 15 min	-0.7725	0.440

HBP, hepatobiliary phases.

**Table 3:** The interobserver reproducibility of the most predictive features on HBP 15 min.

Radiomics parameter	ICC
MedianAbsoluteDeviation	0.924
5Percentile	0.903
Mass	0.997
SphericalDisproportion	0.847
4.7AutoCorrelation	0.898
1.7Contrast	0.850
9.4Contrast	0.801
6.1DifferenceEntropy	0.948
4.7Dissimilarity	0.939
8.4InverseDiffNorm	0.832
1.1InverseVariance	0.975
11.4InverseVariance	0.812
12.4InverseVariance	0.838
8.4MaxProbability	0.929

ICC, intraclass correlation coefficient.

## Data Availability

All data used during the study are available in the article and can be solicited from the corresponding author.
